# Detection of West Nile and Usutu Virus RNA in Autumn Season in Wild Avian Hosts in Northern Italy

**DOI:** 10.3390/v15081771

**Published:** 2023-08-20

**Authors:** Carmela Musto, Marco Tamba, Mattia Calzolari, Arianna Rossi, Annalisa Grisendi, Katia Marzani, Paolo Bonilauri, Mauro Delogu

**Affiliations:** 1 Department of Veterinary Medical Sciences, University of Bologna, 40064 Bologna, Italy; mauro.delogu@unibo.it; 2 Istituto Zooprofilattico Sperimentale della Lombardia e dell’Emilia-Romagna, 25124 Brescia, Italy; marco.tamba@izsler.it (M.T.); mattia.calzolari@izsler.it (M.C.); ariannarossivet@gmail.com (A.R.); annalisa.grisendi@izsler.it (A.G.); katia.marzani@izsler.it (K.M.); paolo.bonilauri@izsler.it (P.B.)

**Keywords:** epidemiological monitoring, flavivirus, off-season monitoring, RNA detection, usutu virus, west nile virus, wild bird

## Abstract

West Nile (WNV) and Usutu (USUV) viruses are two mosquito-borne viruses belonging to the family *Flaviviridae* and genus Flavivirus. The natural transmission cycle of WNV and USUV involves mosquitoes and birds, while mammals are thought to be accidental hosts. The goal of this study was to report—in the context of “off-season monitoring” and passive surveillance—the detection of WNV and USUV RNA in wild birds. To this end, we analyzed biological samples of wild birds in Northern Italy, from October to May, hence outside of the regional monitoring period (June-September). The virological investigations for the detection of USUV and WNV RNA were performed using real-time PCR on frozen samples of the brain, myocardium, kidney, and spleen. In a total sample of 164 wild birds belonging to 27 different species, sequences of both viruses were detected: four birds (2.44%) were positive for WNV and five (3.05%) for USUV. Off-season infections of WNV and especially USUV are still widely discussed and only a few studies have been published to date. To the best of our knowledge, this study is the first report on the detection of USUV RNA until December 22nd. Although further studies are required, our results confirm the viral circulation out-of-season of Flavivirus in wild birds, suggesting reconsidering the epidemiological monitoring period based on each individual climate zone and taking into consideration global warming which will play an important role in the epidemiology of vector-borne diseases.

## 1. Introduction

Usutu (USUV) and West Nile (WNV) viruses are neurotropic mosquito-borne flaviviruses, members of the Japanese encephalitis antigenic group [1]. These two flaviviruses probably have the same life cycles, including ornithophilic mosquitoes (mainly *Culex* spp.) as the main vectors and wild birds as the main amplifying hosts, while mammals are dead-end hosts [2]. West Nile virus (WNV) is nowadays the most widespread virus among the flaviviruses reported in almost all continents. During the last two decades, its presence has been confirmed in several European and Mediterranean countries [3] becoming endemic in some of them. Usutu virus (USUV) was originally isolated in South Africa and detected for the first time in Europe in 2001 where it caused severe mortality in the Austrian Blackbird populations [4]. In the following years, USUV spread to Central and Eastern Europe [5] affecting wild birds including populations from Italy where the virus has probably been circulating since 1996 [6]. USUV was also first detected in the UK in 2020 [5]. In Europe, WNV and USUV appear to be pathogenic for several bird species, especially those in the Passeriformes order, such as jays, crows [7], and birds of prey for WNV [8] and blackbirds and great grey owls (order Strigiformes) for USUV [9,10,11]. As USUV and WNV are zoonotic viruses, regional surveillance programs are necessary for the early detection of their circulation [12]. Clinical cases of neuroinvasive disease and WNV fever, as well as seroconversion in blood donors, were reported in humans [13].

The long-term persistence of arbovirus infection within vertebrate reservoir hosts is a possible mechanism for the overwintering of these viruses at temperate latitudes [14,15]. Viral persistence, defined as the continued presence of infectious viruses in vertebrate hosts beyond the acute viraemic stage, has been documented for most families of arboviruses [16]. This phenomenon may have implications for the maintenance and re-initiation of virus transmission cycles in nature. In temperate regions, extended periods of mosquito inactivity interfere with the continuous transmission of mosquito-borne viruses [17]. Overwintering strategies for these viruses include hibernation of infected adult female mosquitoes, transovarial transmission from female mosquitoes to their offspring, reintroduction through the movement of infectious vectors and/or vertebrate amplifying hosts from warmer climates [18] also if the latter have not yet been directly linked to WNV and USUV overwintering strategy.

As outlined in the National Plan for Prevention Surveillance, and Response to Arbovirus 2020–2025 [19], Italy is divided into three areas: (a) high-risk areas where these Flaviviruses are endemic; (b) low-risk areas where Flaviviruses have been observed only sporadically; and (c) minimum-risk areas where Flaviviruses has never been reported. The Emilia-Romagna region is currently classified as a high-risk area. Migratory and stationary wild birds play an important role in the maintenance and spreading of viruses [2]. The study area has a temperate subcontinental climate and includes three provinces within the Emilia-Romagna region, among them the largest endemic areas in Italy, both for USUV and WNV [20,21]. Thanks to their geographical features, the coexistence of mosquitoes, and stationary and migratory wild birds, these areas provide an ideal environment for epidemiological studies to optimize USUV and WNV surveillance programs [22,23]. In the study area, in the sampling year (2018), the first freezing temperatures occurred in early December [24].

The detection of infectious WNV from the brain, spleen, kidney, and myocardium of an adult female goshawk (*Accipiter gentilis*) in Italy in January—a period of mosquito inactivity—raised questions as to a potential persistent infection within the goshawk or oral transmission to the goshawk via consumption of persistently infected prey, possibly a rodent or bird [25]. Further evidence of a non-mosquito source of transmission during cold periods in a temperate region (New York, NY, USA) was the detection of lethal infections among communally roosting crows [26] and a red-tailed hawk (*Buteo jamaicensis*) [27]. As for USUV, the autumn and winter detection are not reported in the literature but there was a detection during the spring season (April) [28].

In a study by Nemeth et al. [15], it was observed that infectious virus in the house sparrow (*Passer domesticus*)—an established amplifying host for WNV and other arboviruses—persisted in tissues throughout 43 days, while the viral RNA persisted in tissues throughout 65 days, suggesting that such infections in house sparrows could be important for overwintering of WNV. Nemeth et al. [15] hypothesized that there are two overwintering mechanisms in vertebrate reservoir hosts: blood-borne infection of arthropod vectors (recrudescence) and oral infection of vertebrate reservoir hosts (ingestion of infected tissues through predation). As shown by Nemeth et al. [29], persistently WNV-infected birds can act as carriers between endemic and disease-free areas. As every year millions of wild birds migrate between Europe and Africa, they can act as an entry portal, by overwintering in or passing through, and WNV/USUV-endemic areas [30,31,32].

In order to establish whether the circulation of Flaviviruses in wild birds in the Emilia-Romagna region also occurs outside the regional passive surveillance program, which takes place every year from June to September, we conducted this study from October to May. The study’s goals were: (i) to test the detection of WNV RNA in its amplifying hosts in the period off-season of regional surveillance, (ii) to test the detection of USUV RNA in its amplifying hosts in the period off-season of regional surveillance, (iii) to increase the knowledge of the epidemiology of Flavivirus and their possible effects on hosts and the ecosystem.

## 2. Materials and Methods

### 2.1. Birds

**Figure 1:**
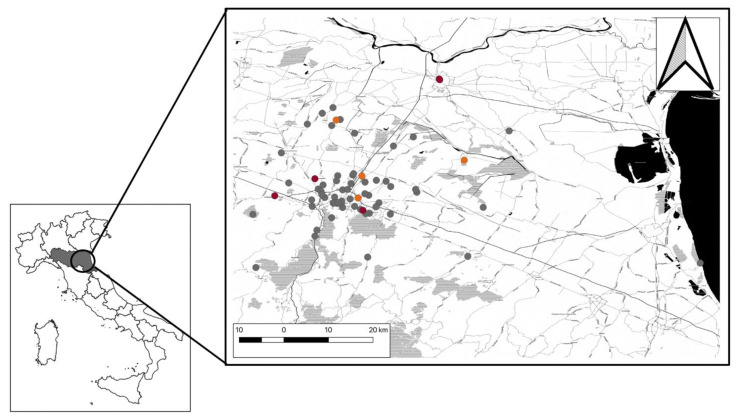
Map of collected wild birds (*n* = 164) in the study area. Red dots indicate birds tested positive for USUV RNA, orange dots indicate WNV RNA positive, and grey dots indicate Flavivirus-negative birds. The position of the Emilia-Romagna region is in the lower-left corner of the figure.

The study involved 164 wild birds (Table 1) collected between October 2018 to May 2019 in the province of Bologna, Ferrara, and Ravenna (Figure 1). The study area map was created using the open-source software Quantum Geographic Information (QGIS^®^) v. 3.10.

The wild birds included in this study were taken from the environment because they showed signs of illness or trauma and were brought to a wildlife rescue center for medical care. Animals that died during the transport or at the rescue center were frozen at −20 °C and delivered—within a maximum of 7 days—to the Department of Veterinary Medical Sciences of the University of Bologna.

Upon the arrival of each bird, a form containing the identification number of the subject, date of collection, coordinates of the place of collection, nutrition status, and age class, was filled out. Age was estimated by body development, observation of plumage, and recorded using the Euring code (Table 2). After external inspection for detecting anomalies or/and lesions, the carcass was positioned in the dorsal position: the subcutis was unglued to examine the pectoral muscles and evaluate the nutritional status (Table 2). Next, the coelomic cavity was opened, exposing the thoracic and abdominal cavities. After inspecting the cavities and the condition of the internal organs, sex was determined by inspecting the gonads (Table 2). For some young subjects (class 1J) with immature gonads, it was not possible to record the sex, thus they were identified as “not determinable” (“ND”). After this investigation, the spleen, kidney, intestines, and myocardium were collected according to the guidelines of the Epidemiological Surveillance of the Emilia-Romagna region and stored at −80 °C until the biomolecular tests. After observing the potential gross pathological findings on the brain structures, the autopsy was completed by sampling the brain and storing it at −80 °C until further testing. The brain, myocardium, kidney, and spleen were tested for the detection of WNV and USUV genomes. Statistical analyses were implemented using the statistical software R (R Core Team 2021) [33]. 

### 2.2. Biomolecular Tests

The organ samples (myocardium, brain, kidney, and spleen) were pooled and tissue specimens were homogenized in 25 mL of PBS in a Stomacher Lab-Blender 80 mL (INTERSCIENCE-France) for 2 min.

Viral RNA was extracted from tissue homogenate in 96 well-plates using the BioSprint^®^ 96 One-For-All Vet kit (Qiagen) and the BioSprint 96 workstation (Qiagen) according to the manufacturer’s instructions. The RNA extracted was tested by real-time RT-PCRs to detect WNV [34,35] and USUV RNAs [36]. The samples that tested positive were tested further with traditional PCRs to obtain amplicons for sequencing: they were submitted to a Pan-flavivirus protocol targeting the NS5 gene [37] and to two specific protocols directed at gene E of WNV [38] and USUV [10]. The amplicons were Sanger sequenced, aligned, and inspected to detect mutations and then utilized for a BLAST search (https://blast.ncbi.nlm.nih.gov/Blast.cgi (accessed on 20 July 2023)).

## 3. Results

Four birds (2.44%) were positive for WNV RNA and five (3.05%) were positive for USUV (Table 1). Of the four WNV-positive birds, three were kestrels and one magpie (Table 1).

All four subjects died in November, came from the province of Bologna, were female, and belonged to age class 3 (Table 2). 

Two of the kestrels and the magpie were in good body condition while the third kestrel was in fair body condition (Supplementary information; Database S1).

Of the five USUV-positive birds, two were blackbirds, one jay, one song thrush, and one scops owl (Table 1). The scops owl died in October, one of the blackbirds in November, the song thrush, the jay, and the other blackbird in December (Supplementary Database S1). The scops owl, a blackbird, and the jay came from the province of Bologna while the thrush and the other blackbird came from the province of Ferrara. The two blackbirds and the song thrush were male while the scops owl and the jay were female, the scops owl, a blackbird, and the jay belonged to age class 3, while the thrush and the other blackbird belonged to age class 4 (Supplementary Database S1). Lastly, all were in good body condition except the scops owl which was cachectic and with polytrauma to the skeletal system. The positive subjects to Flavivirus were in good (7/9-77.8%) nutritional status and the most represented age class was class 3 (7/9-77.8%).

The time spent between entry to the recovery center and the death of the Flavivirus-positive wild birds was 21.3 ± 19.7 h (mean ± standard deviation) with a median of 12 h.

The sampling for the Flavivirus detection was equally distributed among months (Table 2), with a peak in May (43/164-26.22%), the month in which many nestlings require veterinary care. It was equally distributed among sex classes, with 47.56% of males and 43.90% of females (Table 2). The province that provides the most samples was Bologna (87.19%) and the most commonly represented age class was class 4 (67/164-40.85%), consisting of subjects hatched before the current calendar year, but with the exact year unknown, followed by class 6 (50/164-30.49%) representing subjects that certainly hatched before the last calendar year (e.g., many adults in spring), and class 1J (25/164-15.24%) consisting of fledged birds flying so weakly that they could not have flown far from the nest. Lastly, birds from class 3 (22/164-13.41%) had hatched during the current calendar year (Table 2). Additionally, most of the blackbirds were in good (97/164-9.15%) and fair (53/164-32.32%) nutritional status, with only fourteen subjects (8.53%) classified as cachectic (Table 2).

In the four positive birds, the Ct values obtained by the WNV real-time PCRs were 29.8, 37.06, and 37.55 for the European Kestrel and 30.29 for Eurasian Magpie. In the five positive birds, the Ct values obtained by the USUV real-time PCRs were 27.4 and 38.13 for the Eurasian Blackbirds, 37.71 for the Song Thrush, 31.96 for the Eurasian jay, and 38.12 Eurasian Scops Owl. Moreover, we obtained two amplicon sequences from the USUV (one from Eurasian Blackbirds and one from Eurasian Jay). These two sequences differed by one mismatch on the 408 nucleotides of the sequences and showed an identity (ranging from complete to one mismatch) sequences obtained from mosquitoes and a Eurasian Collared Dave (*Streptopelia decaocto*) in Emilia-Romagna region in 2010 (GB: JF834599, JF834604, JF834606, JF834616, JF834623, JF834626, JF834657, and JF834673). We also obtained two amplicon sequences from WNV, one from a European Kestrel and one from the Eurasian Magpie, which showed three mutations on the 392 nucleotides of the amplicons. Both the sequences obtained from WNV showed the highest identity (showing from one to four mismatches) for two sequences obtained in Italy from mosquitoes in 2013 (GB KU573083, KU573080) and two sequences of human origin from Austria obtained in 2014 and 2015 (KP109692, MF984340). Interestingly these sequences, belonging to lineage 2 of WNV, shared the same mutation concerning the other sequences deposed in GeneBank.

## 4. Discussion

Here, we present the results of passive surveillance for WNV and USUV detection, covering wild birds during the autumn–winter–spring period, during which the circulation of their main vectors is strongly reduced or absent.

On a sample of 164 wild birds belonging to 27 species, both genomes were found, four birds (2.44%) were positive for WNV and five (3.05%) for USUV. Our findings confirm the presence of WNV and USUV RNA in subjects belonging mainly to the Order of Passeriformes [39], Falconiformes, and Strigiformes [40], demonstrating that these three orders are among the main amplifying hosts of *Flaviviridae*, with some species highly susceptible, as in the case of blackbirds with USUV [20]. We could not analyze fresh subjects with cytological and histopathological techniques, and we do not know if the animals were viremic and for how long they had been before their death. The Ct value obtained by real-time PCR is not a precise estimation of the viral load present, but it gives an indication, with fewer cycles relating to higher amounts of virus. Applying an arbitrary threshold around Ct 30, we can classify birds under this threshold as bearing a high load of viruses, and birds over this threshold as bearing a low load of viruses. According to this classification, one blackbird showed a high amount of USUV, while the other birds had a low amount. This result supports the primary role of blackbirds as an amplifier host of USUV. Birds with a high load of WNV were a kestrel and a magpie, supporting the idea of several different bird species being able to act as amplifier hosts for this virus, for which a key bird species was not identified.

The detection of the viral RNA in these tissues in the off-season period of regional surveillance requires some considerations. The first aspect that needs to be clarified is the time of infection: to this end, several questions should be asked. (1) Is it the case that subjects became infected in the warmer months and were positive from October until December?; (2) did they become infected during the cold months or even shortly before their death?

Regarding the first question, our study found positive subjects from October 16th until December 22nd. Nemeth et al. [15] observed that viral RNA in Passeriformes persisted in tissues for 65 days, this could explain the detection of positive animals between October and early December. Indeed, in other temperate areas such as Spain, outbreaks in horses usually occur until the last weeks of November [41] and in temperate areas—as in the case of Italy—mosquitoes are active from May to early October [42]. Due to global warming, it has become common to observe higher temperatures in the autumn months in temperate climates [43] demonstrating that climatic changes will play an important role in the epidemiology of vector-borne diseases [44,45].

Regarding the second consideration, for predatory birds, the transmission of WNV through predation on infected prey has been frequently observed [40,46]. However, although this provides a possible explanation of how kestrels and scops owls could have obtained the infection, it does not explain how the birds that were preyed upon could still have been infectious in a period of low mosquito activity. An alternative could be that virus acquisition occurred through predating birds with long-term, persistent infection. The persistence of infectious WNV for prolonged periods in the organs of birds has been confirmed by many authors [27,47,48,49,50,51]. Persistent infection is defined as the detection of a virus in host tissues after viremia has subsided [52]. The persistent viral loads in the organs of birds belonging to prey species might sustain flaviviruses transmission to predators after the end of the mosquito season. The recent finding of WNV Lineage2 in an adult female goshawk [25] and in a little grebe [14] collected in the winter months in the Umbria region (Italy) indicates that detection of birds with WNV-infected organs in the winter months can be possible [15,40,50,51,52]. Persistent infection is a hypothesis that needs to be investigated and explored but due to the limitations of the study, it is not possible to endorse or deny this hypothesis, since it is not known how long the birds were positive, although, in the case of the European blackbird with USUV RNA on December 22nd, the persistent infection hypothesis appears the most plausible.

Bearing in mind that common prey species of predatory birds include rodents and that, also in these animals, infectious WNV has been detected months after infection [53,54], rodent-to-bird transmission appears plausible as well. As reported by Mencattelli et al. [25], further research is needed to better understand the transmission routes and the role of overwintering birds, rodents, and mosquitoes, as well as WNV and USUV infection per *os*.

Alternatively, the infection could have happened in the wildlife rescue center through contact with infected, still viremic birds during the summer. However, the authors of this work believe that this cannot be supported since the mean permanence of a bird was 21.3 ± 19.7 h (mean ± standard deviation).

In outlining the possible hypotheses, we must consider that in 2018, there was an exceptional and very intense circulation of WNV throughout Europe [55,56] and it is possible that the high number of subjects still positive in autumn 2018–2019 depended on the fact that in the summer season, they were subjected to an extraordinarily high infectious pressure.

Assessing the causes of death of wild birds has been shown to be a useful practice to detect virus circulation [9] and should be maintained and encouraged also in the autumn–winter period, due to the importance of the health surveillance of these potentially zoonotic viruses. Most of the subjects had collision trauma but we cannot establish whether this was a consequence of the Flavivirus infection or a coincidental finding. By undermining the functionality of the neural system, Flavivirus might increase the risk of bird collisions with the surrounding environment and anthropogenic elements (e.g., buildings and cars) [9]. Most of the positive subjects were in a good nutritional state (7/9-77.8%), which could suggest asymptomatic positivity or acute virosis.

The obtained field sequences, even if short, showed high similarity to isolated sequences from the same geographical area, suggesting the overwintering of the same strains [22]. These data are in line with those reported recently on the genomic evolution of WNV and USUV in Italy and Europe. Thus, results further confirm that the circulation of WNV and USUV in Italy and Europe is mainly determined by the inner circulation of strains evolved locally, which are able to expand to other areas and just to a lesser extent by external influxes [30,57,58].

We reported a preliminary study from a limited area using a convenience sample. This approach is commonly adopted for this type of study (e.g., Germany [59]; Austria [60]) but in the case of opportunistic sampling, it is important to highlight that it cannot make any claims about the WNV and USUV incidence and inference in time or space.

## 5. Conclusions

In this study, the last detection for WNV RNA was on November 22nd and for USUV RNA on December 22nd. It is possible that viruses could still circulate in mosquitoes until the beginning of October, while mosquito activity can vary from season to season, after this period the outdoor activity of mosquitoes is strongly unlikely. In fact, the dynamic of *Culex pipiens* in the study area (Emilia-Romagna region) is characterized by the presence of a low number of hosts seeking mosquitoes from May, a peck of density at the end of June and the beginning of July, and a decreasing until the beginning of October, when a low number of mosquitoes are active and West Nile positive pools were collected sporadically [42,61]. Considering that eight out of nine birds tested positive between October 16th and December 3rd, the most plausible hypothesis is that the subjects were infected by mosquitoes in early October and that traces of RNA were still present in the tissues [15]. While for the Eurasian blackbird with USUV RNA at the end of December, the most plausible hypothesis is that could be a case of persistent infection.

As reported cases of Flavivirus positivity remain rare during the autumn season, this report is of particular importance because it confirms the possibility that there is still circulation of the infected vector. The data obtained suggest reconsidering the epidemiological monitoring period based on each individual climate zone, also taking into consideration global warming which will play an important role in the epidemiology of vector-borne diseases, influencing the period of activity of mosquitoes and requesting an extension of the surveillance period.

For the first time, USUV RNA was detected in blackbirds in October, November, and December.

This work, albeit recognizing the limit and statistical biases characterizing passive surveillance, has demonstrated and highlighted the strong importance of monitoring and surveillance of wildlife. This result suggests that the passive monitoring performed through the Regional Plan of Epidemiological Surveillance on recovered wild birds can help better understand these flaviviruses’ epidemiology and their new circulation scenarios.

## Figures and Tables

**Table 1:** Species identification of the population sampled with results of detection of WNV and USUV RNA.

Order	SPECIES Common Name	SPECIES Scientific Name	No. Subjects Tested (*n*-%)	WNV Positive (*n* = 4)	Prev	95% CI	USUV Positive (*n* = 5)	Prev	95% CI
	Common buzzard	*Buteo buteo*	7-4.27%	0	0.00%	40.96%	0	0.00%	40.96%
*Accipitriformes*	Eurasian sparrowhawk	*Accipiter nisus*	2-1.22%	0	0.00%	84.19%	0	0.00%	84.19%
*Apodiformes*	Common swift	*Apus apus*	5-3.04%	0	0.00%	52.18%	0	0.00%	52.18%
*Falconiformes*	European kestrel	*Falco tinnunculus*	11-6.71%	3	27.27%	60.97%	0	0.00%	28.49%
	Eurasian hobby	*Falco subbuteo*	1-0.61%	0	0.00%	97.50%	0	0.00%	97.50%
	Eurasian blackcap	*Sylvia atricapilla*	2-1.22%	0	0.00%	84.19%	0	0.00%	84.19%
	Eurasian great tit	*Parus major*	1-0.61%	0	0.00%	97.50%	0	0.00%	97.50%
	Long-tailed tit	*Aegithalos caudatus*	2-1.22%	0	0.00%	84.19%	0	0.00%	84.19%
	Hooded crow	*Corvus cornix*	2-1.22%	0	0.00%	84.19%	0	0.00%	84.19%
	Common chaffinch	*Fringilla coelebs*	1-0.61%	0	0.00%	97.50%	0	0.00%	97.50%
	Eurasian magpie	*Pica pica*	18-10.97%	1	5.55%	27.29%	0	0.00%	18.53%
	Eurasian jay	*Garrulus glandarius*	12-7.32%	0	0.00%	26.46%	1	8.33%	38.48%
*Passeriformes*	Eurasian blackbird	*Turdus merula*	43-26.22%	0	0.00%	8.22%	2	4.65%	15.81%
	House sparrow	*Passer domesticus*	2-1.22%	0	0.00%	84.19%	0	0.00%	84.19%
	European robin	*Erithacus rubecula*	15-9.15%	0	0.00%	21.80%	0	0.00%	21.80%
	Goldcrest	*Regulus regulus*	1-0.61%	0	0.00%	97.50%	0	0.00%	97.50%
	European starling	*Sturnus vulgaris*	11-6.71%	0	0.00%	28.49%	0	0.00%	28.49%
	Song thrush	*Turdus philomelos*	5-3.04%	0	0.00%	52.18%	1	20.00%	71.64%
	European greenfinch	*Chloris chloris*	1-0.61%	0	0.00%	97.50%	0	0.00%	97.50%
	Grey heron	*Ardea cinerea*	3-1.83%	0	0.00%	70.75%	0	0.00%	70.75%
*Pelecaniformes*	Cattle egret	*Bubulcus ibis*	1-0.61%	0	0.00%	97.50%	0	0.00%	97.50%
	Little egret	*Egretta garzetta*	1-0.61%	0	0.00%	97.50%	0	0.00%	97.50%
	Eurasian tawny owl	*Strix aluco*	4-2.44%	0	0.00%	60.23%	0	0.00%	60.23%
	Eurasian scops owl	*Otus scops*	4-2.44%	0	0.00%	60.23%	1	25.00%	80.59%
*Strigiformes*	Barn owl	*Tyto alba*	1-0.61%	0	0.00%	97.50%	0	0.00%	97.50%
	Little owl	*Athene noctua*	7-4.27%	0	0.00%	40.96%	0	0.00%	40.96%
	Long-eared owl	*Asio otus*	1-0.61%	0	0.00%	97.50%	0	0.00%	97.50%
Tot. order: 6	Tot. species: 27		164-100%	4 (2.44%)			5 (3.05%)		

Abbreviations: *n*: number; Prev: prevalence; 95% CI: upper limit of the confidence interval.

**Table 2:** Signaling data of the population sampled with results of molecular investigations of WNV and USUV according to the month of death, province of origin, sex, and age.

	No. Subjects (*n*-%)	WNV Positive (*n* = 4)	Prev	95% CI	USUV Positive (*n* = 5)	Prev	95% CI
**Month of sampling**							
October	17-10.36%	0	0.00%	19.51%	1	5.88%	28.69%
November	19-11.58%	4	21.05%	45.56%	1	5.26%	26.03%
December	29-17.68%	0	0.00%	11.94%	3	10.34%	27.35%
January	10-6.10%	0	0.00%	30.85%	0	0.00%	30.85%
February	14-8.54%	0	0.00%	23.16%	0	0.00%	23.16%
March	19-11.59%	0	0.00%	17.65%	0	0.00%	17.65%
April	13-7.93%	0	0.00%	24.70%	0	0.00%	24.70%
May	43-26.22%	0	0.00%	8.22%	0	0.00%	8.22%
**Province**							
Bologna	143-87.19%	4	2.80%	7.00%	3	2.10%	6.00%
Ferrara	7-4.27%	0	0.00%	40.96%	2	28.57%	70.96%
Ravenna	14-8.53%	0	0.00%	23.16%	0	0.00%	23.16%
**Sex**							
Male	78-47.56%	0	0.00%	4.62%	3	3.85%	10.83%
Female ND	72-43.90% 14-8.53%	4 0	5.55% 0.00%	13.62% 23.16%	2 0	2.78% 0.00%	9.68% 23.16%
**Euring age codes**							
Class_1J	25-15.24%	0	0.00%	13.72%	0	0.00%	13.72%
Class_3	22-13.41%	4	18.18%	40.28%	3	13.64%	34.91%
Class_4	67-40.85%	0	0.00%	5.36%	2	2.98%	10.37%
Class_6	50-30.49%	0	0.00%	7.11%	0	0.00%	7.11%
**Body condition**							
Good	97-59.15%	3	3.09%	8.77%	4	4.12%	10.22%
Fair	53-32.32%	1	1.89%	10.07%	0	0.00%	6.72%
Cachectic	14-8.53%	0	0.00%	23.16%	1	7.14%	33.87%

Abbreviations: *n*: number; ND: not determinable; Prev: prevalence; 95% CI: upper limit of the confidence interval. Euring age codes: 1J: fledged but flying so weakly that it is incapable of having flown far from the nest; 3: Definitely hatched during current calendar year; 4: Hatched before current calendar year—exact year unknown; 6: Hatched before last calendar year—exact year unknown (e.g., many adults in spring).

## Data Availability

Not applicable.

## Supplementary Materials

The following supporting information can be downloaded at: https://www.mdpi.com/article/10.3390/v15081771/s1, Database S1: sampling dataset associated with the “ReadMe” file showing the explanation of the variables reported in the dataset.

## References

[1] Bakonyi T., Gould E.A., Kolodziejek J., Weissenbck H., Nowotny N. (2004). Complete genome analysis and molecular characterization of Usutu virus that emerged in Austria in 2001: Comparison with the South African strain SAAR-1776 and other Flaviviruses. Virology.

[2] Zannoli S., Sambri V. (2019). West Nile Virus and Usutu Virus Co-Circulation in Europe: Epidemiology and Implications. Microorganisms.

[3] Rizzoli A., Jiménez-Clavero M.A., Barzon L., Cordioli P., Figuerola J., Koraka P., Martina B., Moreno A., Nowotny N., Pardigon N. (2015). The challenge of West Nile virus in Europe: Knowledge gaps and research priorities. Eurosurveillance.

[4] Chvala S., Kolodziejek J., Nowotny N., Weissenböck H. (2004). Pathology and Viral Distribution in Fatal Usutu Virus Infections of Birds from the 2001 and 2002 Outbreaks in Austria. J. Comp. Pathol..

[5] Folly A.J., Lawson B., Lean Fabian Z.X., McCracken F., Spiro S., John Shinto K., Heaver J.P., Seilern-Moy K., Masters N., Hernández Triana L.M. (2020). Detection of Usutu virus infection in wild birds in the United Kingdom, 2020. Euro Surveill..

[6] Weissenböck H., Bakonyi T., Rossi G., Mani P., Nowotny N. (2013). Usutu virus, Italy, 1996. Emerg. Infect. Dis..

[7] Perez-Ramirez E., Llorente F., Jimenez-Clavero M.A. (2014). Experimental Infections of Wild Birds with West Nile Virus. Viruses.

[8] Ziegler U., Angenvoort J., Fischer D., Fast C., Eiden M., Rodriguez A.V., Revilla-Fernández S., Nowotny N., de la Fuente J.G., Lierz M. (2013). Pathogenesis of West Nile virus lineage 1 and 2 in experimentally infected large falcons. Vet. Microbiol..

[9] Musto C., Tamba M., Calzolari M., Torri D., Marzani K., Cerri J., Bonilauri P., Delogu M. (2022). Usutu virus in blackbirds (*Turdus merula*) with clinical signs, a case study from northern Italy. Eur. J. Wildl. Res..

[10] Manarolla G., Bakonyi T., Gallazzi D., Crosta L., Weissenböck H., Dorrestein G.M., Nowotny N. (2010). Usutu virus in wild birds in northern Italy. Vet. Microbiol..

[11] Weissenböck H., Kolodziejek J., Fragner K., Kuhn R., Pfeffer M., Nowotny N. (2003). Usutu Virus Activity in Austria, 2001–2002. Microbes. Infect..

[12] Domanovic D., Gossner C.M., Lieshout-Krikke R., Mayr W., Baroti-Toth K., Dobrota A.M., Escoval M.A., Henseler O., Jungbauer C., Liumbruno G. (2019). West Nile and Usutu Virus Infections and Challenges to Blood Safety in the European Union. Emerg. Infect. Dis..

[13] Petersen L.R., Roehrig J.T. (2001). West Nile Virus: A Reemerging Global Pathogen. Emerg. Infect. Dis..

[14] Giglia G., Mencattelli G., Lepri E., Agliani G., Gobbi M., Gröne A., van den Brand J.M.A., Savini G., Mandara M.T. (2022). West Nile Virus and Usutu Virus: A Post-Mortem Monitoring Study in Wild Birds from Rescue Centers, Central Italy. Viruses.

[15] Nemeth N., Young G., Ndaluka C., Bielefeldt-Ohmann H., Komar N., Bowen R. (2009). Persistent West Nile virus infection in the house sparrow (*Passer domesticus*). Arch. Virol..

[16] Kuno G. (2001). Persistence of arboviruses and antiviral antibodies in vertebrate hosts: Its occurrence and impacts. Rev. Med. Virol..

[17] Reisen W.K., Chiles R.E., Green E.N., Fang Y., Mahmood F. (2003). Previous infection protects house finches from re-infection with St. Louis encephalitis virus. J. Med. Entomol..

[18] Reeves W.C., Reeves W.C. (1990). Overwintering of arboviruses. Epidemiology and Control of Mosquito-Borne Arboviruses in California, 1943–1987.

[19] National Plan for Prevention, Surveillance, and Response to Arbovirus 2020–2025 (2020). https://www.salute.gov.it/imgs/C_17_pubblicazioni_2947_allegato.pdf.

[20] Lauriano A., Rossi A., Galletti G., Casadei G., Santi A., Rubini S., Carra E., Lelli D., Calzolari M., Tamba M. (2021). West Nile and Usutu Viruses’ Surveillance in Birds of the Province of Ferrara, Italy, from 2015 to 2019. Viruses.

[21] Tamba M., Bonilauri P., Bellini R., Calzolari M., Albieri A., Sambri V., Dottori M., Angelini P. (2011). Detection of Usutu Virus within a West Nile Virus Surveillance Program in Northern Italy. Vector Borne Zoonotic Dis..

[22] Calzolari M., Chiapponi C., Bonilauri P., Lelli D., Baioni L., Barbieri I., Lavazza A., Pongolini S., Dottori M., Moreno A. (2017). Co-Circulation of Two Usutu Virus Strains in Northern Italy between 2009 and 2014. Infect. Genet. Evol..

[23] Calzolari M., Bonilauri P., Bellini R., Albieri A., Defilippo F., Maioli G., Galletti G., Gelati A., Barbieri I., Tamba M. (2010). Evidence of Simultaneous Circulation of West Nile and Usutu Viruses in Mosquitoes Sampled in Emilia-Romagna Region (Italy) in 2009. PLoS ONE.

[24] World Weather Online. https://www.worldweatheronline.com/bologna-weather-history/emilia-romagna/it.aspx.

[25] Mencattelli G., Iapaolo F., Polci A., Marcacci M., Di Gennaro A., Teodori L., Curini V., Di Lollo V., Secondini B., Scialabba S. (2022). West Nile Virus Lineage 2 Overwintering in Italy. Trop. Med. Infect. Dis..

[26] Dawson J.R., Stone W.B., Ebel G.D., Young D.S., Galinski D.S., Pensabene J.P., Franke M.A., Eidson M., Kramer L.D. (2007). Crow deaths caused by West Nile virus during winter. Emerg. Infect. Dis..

[27] Garmendia A.E., Van Kruiningen H.J., French R.A., Anderson J.F., Andreadis T.G., Kumar A., West B. (2000). Recovery and identification of West Nile virus from a hawk in winter. J. Clin. Microbiol..

[28] Rijks J., Kik M., Slaterus R., Foppen R., Stroo A., IJzer J., Stahl J., Gröne A., Koopmans M., van der Jeugd H. (2016). Widespread Usutu virus outbreak in birds in the Netherlands, 2016. Euro Surveill..

[29] Nemeth N.M., Oesterle P.T., Bowen R.A. (2009). Humoral Immunity to West Nile Virus is Long-Lasting and Protective in the House Sparrow (*Passer domesticus*). Am. J. Trop. Med. Hyg..

[30] Mancuso E., Cecere J.G., Iapaolo F., Di Gennaro A., Sacchi M., Savini G., Spina F., Monaco F. (2022). West Nile and Usutu Virus Introduction via Migratory Birds: A Retrospective Analysis in Italy. Viruses.

[31] Rappole J.H., Hubalek Z. (2003). Migratory Birds and West Nile Virus. J. Appl. Microbiol..

[32] Rappole J.H., Derrickson S.R., Hubalek Z. (2000). Migratory Birds and Spread of West Nile Virus in the Western Hemisphere. Emerg. Infect. Dis..

[33] R Core Team (2020). R: A Language and Environment for Statistical Computing. R Foundation for Statistical Computing. Vienna, Austria. https://www.r-project.org/.

[34] Tang Y., Anne Hapip C., Liu B., Fang C.T. (2006). Highly sensitive TaqMan RT-PCR assay for detection and quantification of both lineages of West Nile virus RNA. J. Clin. Virol..

[35] Del Amo J., Sotelo E., Fernández-Pinero J., Gallardo C., Llorente F., Agüero M., Jiménez-Clavero M.A. (2013). A novel quantitative multiplex real-time RT-PCR for the simultaneous detection and differentiation of West Nile virus lineages 1 and 2, and of Usutu virus. J. Virol. Methods.

[36] Cavrini F., Della Pepa M.E., Gaibani P., Pierro A.M., Rossini G., Landini M.P., Sambri V. (2011). A rapid and specific real-time RT-PCR assay to identify usutu virus in human plasma, serum, and cerebrospinal fluid. J. Clin. Virol..

[37] Scaramozzino N., Crance J.M., Jouan A., DeBriel D.A., Stoll F., Garin D. (2001). Comparison of Flavivirus universal primer pairs and development of a rapid, highly sensitive heminested reverse transcription-PCR assay for detection of Flaviviruses targeted to a conserved region of the NS5 gene sequences. J. Clin. Microbiol..

[38] Lanciotti R.S., Kerst A.J., Nasci R.S., Godsey M.S., Mitchell C.J., Savage H.M., Komar N., Panella N.A., Allen B.C., Volpe K.E. (2000). Rapid detection of west nile virus from human clinical specimens, field-collected mosquitoes, and avian samples by a TaqMan reverse transcriptase-PCR assay. J. Clin. Microbiol..

[39] Roiz D., Vázquez A., Ruiz S., Tenorio A., Soriguer R., Figuerola J. (2019). Evidence that Passerine Birds Act as Amplifying Hosts for Usutu Virus Circulation. EcoHealth.

[40] Vidaña B., Busquets N., Napp S., Pérez-Ramírez E., Jiménez-Clavero M.Á., Johnson N. (2020). The Role of Birds of Prey in West Nile Virus Epidemiology. Vaccines.

[41] García-Bocanegra I., Belkhiria J., Napp S., Cano-Terriza D., Jiménez-Ruiz S., Martínez-López B. (2018). Epidemiology and spatio-temporal analysis of West Nile virus in horses in Spain between 2010 and 2016. Transbound. Emerg. Dis..

[42] Carrieri M., Fariselli P., Maccagnani B., Angelini P., Calzolari M., Bellini R. (2014). Weather Factors Influencing the Population Dynamics of *Culex pipiens* (Diptera: Culicidae) in the Po Plain Valley, Italy (1997–2011). Environ. Entomol..

[43] Lakson M., Post P., Sepp M. (2019). The Impact of Atmospheric Circulation on Air Temperature Rise in Estonia. Front. Earth Sci..

[44] Yi H., Devkota B.R., Yu J.S., Oh K.C., Kim J., Kim H.J. (2014). Effects of global warming on mosquitoes & mosquito-borne diseases and the new strategies for mosquito control. Entomol. Res..

[45] Reeves W.C., Hardy J.L., Reisen W.K., Milby M.M. (1994). Potential Effect of Global Warming on Mosquito-Borne Arboviruses. J. Med. Entomol..

[46] Nemeth N., Gould D., Bowen R., Komar N. (2006). Natural and Experimental West Nile Virus Infection in Five Raptor Species. J. Wildl. Dis..

[47] Montecino-Latorre D., Barker C.M. (2018). Overwintering of West Nile Virus in a Bird Community with a Communal Crow Roost. Sci. Rep..

[48] Conte A., Candeloro L., Ippoliti C., Monaco F., Massis F.D., Bruno R., Sabatino D.D., Danzetta M.L., Benjelloun A., Belkadi B. (2015). Spatio-Temporal Identification of Areas Suitable for West Nile Disease in the Mediterranean Basin and Central Europe. PLoS ONE.

[49] Wheeler S.S., Vineyard M.P., Woods L.W., Reisen W.K. (2012). Dynamics of West Nile Virus Persistence in House Sparrows (*Passer Domesticus*). PLoS Negl. Trop. Dis..

[50] Reisen W.K., Fang Y., Lothrop H.D., Martinez V.M., Wilson J., Oconnor P., Carney R., Cahoon-Young B., Shafii M., Brault A.C. (2006). Overwintering of West Nile Virus in Southern California. J. Med. Entomol..

[51] Komar N., Langevin S., Hinten S., Nemeth N., Edwards E., Hettler D., Davis B., Bowen R., Bunning M. (2003). Experimental Infection of North American Birds with the New York 1999 Strain of West Nile Virus. Emerg. Infect. Dis..

[52] Wheeler S.S., Langevin S.A., Brault A.C., Woods L., Carroll B.D., Reisen W.K. (2012). Detection of Persistent West Nile Virus RNA in Experimentally and Naturally Infected Avian Hosts. Am. J. Trop Med. Hyg..

[53] Appler K.K., Brown A.N., Stewart B.S., Behr M.J., Demarest V.L., Wong S.J., Bernard K.A. (2010). Persistence of West Nile Virus in the Central Nervous System and Periphery of Mice. PLoS ONE.

[54] Tesh R.B., Siirin M., Guzman H., Travassos da Rosa A.P.A., Wu X., Duan T., Lei H., Nunes M.R., Xiao S.Y. (2005). Persistent West Nile Virus Infection in the Golden Hamster: Studies on Its Mechanism and Possible Implications for Other Flavivirus Infections. J. Infect. Dis..

[55] Young J.J., Haussig J.M., Aberle S.W., Pervanidou D., Riccardo F., Sekulić N., Bakonyi T., Gossner C.M. (2021). Epidemiology of human West Nile virus infections in the European Union and European Union enlargement countries, 2010 to 2018. Eur. J. Infect. Dis. Surveill. Epidemiol. Prev. Control..

[56] Camp J.V., Nowotny N. (2020). The knowns and unknowns of West Nile virus in Europe: What did we learn from the 2018 outbreak?. Expert Rev. Anti-Infect. Ther..

[57] Beck C., Goffart I.L., Franke F., Gonzalez G., Dumarest M., Lowenski S., Blanchard Y., Lucas P., Lamballerie X., Grard G. (2020). Contrasted Epidemiological Patterns of West Nile Virus Lineages 1 and 2 Infections in France from 2015 to 2019. Pathogens.

[58] Mencattelli G., Iapaolo F., Monaco F., Fusco G., de Martinis C., Portanti O., Di Gennaro A., Curini V., Polci A., Berjaoui S. (2021). West Nile Virus Lineage 1 in Italy: Newly Introduced or a Re-Occurrence of a Previously Circulating Strain?. Viruses.

[59] Becker N., Jöst H., Ziegler U., Eiden M., Höper D., Emmerich P., Fichet-Calvet E., Ehichioya D.U., Czajka C., Gabriel M. (2012). Epizootic emergence of Usutu virus in wild and captive birds in Germany. PLoS ONE.

[60] Chvala S., Bakonyi T., Bukovsky C., Meister T., Brugger K., Rubel F., Nowotny N., Weissenböck H. (2007). Monitoring of Usutu virus activity and spread by using dead bird surveillance in Austria, 2003–2005. Vet Microbiol.

[61] Calzolari M., Angelini P., Bolzoni L., Bonilauri P., Cagarelli R., Canziani S., Cereda D., Cerioli M.P., Chiari M., Galletti G. (2020). Enhanced West Nile Virus Circulation in the Emilia-Romagna and Lombardy Regions (Northern Italy) in 2018 Detected by Entomological Surveillance. Front. Vet. Sci..

