# Supplementary material for: Detection of West Nile and Usutu Virus RNA in Autumn Season in Wild Avian Hosts in Northern Italy
Source: Viruses. 2023 Aug 20;15(8):1771. doi: 10.3390/v15081771 (PMC10458002; doi:10.3390/v15081771)
Supplement: Supplementary file 1 [file viruses-15-01771-s001.zip › viruses-2566741-SI.pdf]

SUPPLEMENTARY MATERIAL

README file

Information

The following dataset it is meant to be a Supplementary Material for the study entitled "**Detection of West Nile and Usutu Virus RNA in Autumn Season in Wild Avian Hosts in Northern Italy**".

Variables reported in the dataset:

- "ID_unique" – identification number of the sampled individual.
- "day_entry" – date of entry of the animals in the wildlife recovery center.
- "day_death" - date of death of animals in wildlife recovery center.
- "month" - month of sampling of wild birds.
- "year" – year of sampling of wild birds.
- "order" – order of the wild birds sampled.
- "species" - species of the wild birds sampled.
- "binomial" – Binomial nomenclature of the wild birds sampled.
- "sex" – sex of the wild birds sampled.
- "age" – estimate of the age of wild birds expressed in "Euring age codes".
- "province" – province of discovery of wild birds.
- "lat" – Latitude.
- "long" – Longitude.
- "WND" – Dichotomous variable indicating if positive or negative WND (1 = "Yes", 0 = "No").
- "USUV" – Dichotomous variable indicating if positive or negative USUV (1 = "Yes", 0 = "No").

- "body_condition" - Dichotomous variable indicating the body condition of the sampling wild birds in the study (1 = “good”, 2 = “fair”, 3 = “cachectic”).
